# Self-Powered Organometal Halide Perovskite Photodetector with Embedded Silver Nanowires

**DOI:** 10.3390/nano12071034

**Published:** 2022-03-22

**Authors:** Almaz R. Beisenbayev, Zhandos T. Sadirkhanov, Yerassyl Yerlanuly, Marat I. Kaikanov, Askhat N. Jumabekov

**Affiliations:** 1 Department of Chemical Engineering, Nazarbayev University, Nur-Sultan 010000, Kazakhstan; almaz.beisenbayev@nu.edu.kz; 2 Department of Physics, Nazarbayev University, Nur-Sultan 010000, Kazakhstan; zhandos.sadirkhanov@nu.edu.kz (Z.T.S.); yerassyl.yerlanuly@nu.edu.kz (Y.Y.); marat.kaikanov@nu.edu.kz (M.I.K.)

**Keywords:** perovskite, silver nanowires, photodetector, network, methylamine

## Abstract

Metal–semiconductor–metal (MSM) configuration of perovskite photodetectors (PPDs) suggests easy and low-cost manufacturing. However, the basic structures of MSM PPDs include vertical and lateral configurations, which require the use of expensive materials such as transparent conductive oxides or/and sophisticated fabrication techniques such as lithography. Integrating metallic nanowire-based electrodes into the perovskite photo-absorber layer to form one-half of the MSM PPD structure could potentially resolve the key issues of both configurations. Here, a manufacturing of solution-processed and self-powered MSM PPDs with embedded silver nanowire electrodes is demonstrated. The embedding of silver nanowire electrode into the perovskite layer is achieved by treating the silver nanowire/perovskite double layer with a methylamine gas vapor. The evaporated gold layer is used as the second electrode to form MSM PPDs. The prepared MSM PPDs show a photoresponsivity of 4 × 10^−5^ AW^−1^ in the UV region and 2 × 10^−5^ AW^−1^ in the visible region. On average, the devices exhibit a photocurrent of 1.1 × 10^−6^ A under white light (75 mW cm^−2^) illumination with an ON/OFF ratio of 83.4. The results presented in this work open up a new method for development and fabrication of simple, solution-processable MSM self-powered PPDs.

## 1. Introduction

Photodetectors (PDs) have been used widely in many commercial and scientific technologies, and have found applications in a number of emerging new fields such as imaging, biomedical sensing, visible-light communication, wearable/portable electronics, etc. [1,2,3,4,5,6,7]. The manufacturing process of PDs based on conventional inorganic semiconductor materials is usually complex and can be costly [7,8]. Moreover, most of these commonly used inorganic materials for PD technology are mechanically rigid, brittle, and opaque, which can limit their application in flexible, semi-transparent, and transparent PD technologies [6,9,10,11,12]. Additionally, the bandgap of most of the traditional inorganic materials used in PD technology is difficult to tune, making them less versatile than some of the organic and organic/inorganic alternative materials proposed in recent years [13]. Therefore, the development of affordable, broadband, and easy-to-manufacture PDs is of great importance.

Recently, organometal halide perovskites have attracted a significant attention in the optoelectronics research community due to their outstanding properties such as low-temperature solution-processability, long charge-carrier diffusion lengths, tunable bandgap, and strong absorption of visible light [14,15,16,17,18,19,20]. Hence, perovskites are promising materials for applications in light-emitting diodes, lasers, and broadband PDs [21,22,23,24,25,26,27]. Despite their exceptional optoelectronic properties, there are a number of factors that can affect the performance of perovskite photodetectors (PPDs). Some of these factors are the perovskite film quality (e.g., uniformity of the perovskite layer and absence of pinholes), degree of polycrystallinity, surface and bulk defect density, stability to environmental factors (e.g., atmosphere, temperature, and humidity), and photostability [28]. One way to address these problems is to use single-crystalline perovskite films for making PPDs [28,29,30,31,32]. Compared to polycrystalline perovskites, single-crystalline perovskites possess better film quality and, thus, afford a higher charge carrier mobility, longer carrier lifetime, and improved stability [28,32]. While the synthesis of single-crystalline perovskite films is often challenging, surface and interface engineering is another route to improve the performance of PPDs employing polycrystalline perovskite photo-absorber layers [33,34,35,36].

In terms of device structure, PPDs can be made from the combinations of perovskites with various semiconductor materials to form type-II diode heterostructures (p–n or p–i–n heterojunctions), or can have a Schottky diode structure. In the latter, various combinations of the perovskite and metallic layers are formed (metal–semiconductor (MS) or metal–semiconductor–metal (MSM) junctions) to construct PPDs [36,37,38,39,40,41,42]. Among these, the MSM configuration proposes a comparatively low-cost and easier manufacturing due to its structural simplicity and absence of the electron- (n-type) and hole-transporting (p-type) layers [36,43,44]. Hence, MSM PPDs offer an effective way to fabricate self-powered PPD devices. The built-in potential in self-powered MSM PPDs, which drives the photogenerated charge carriers to their respective contacts without any applied bias, can be formed by two metallic layers with dissimilar work functions [45,46].

**Figure 1:**
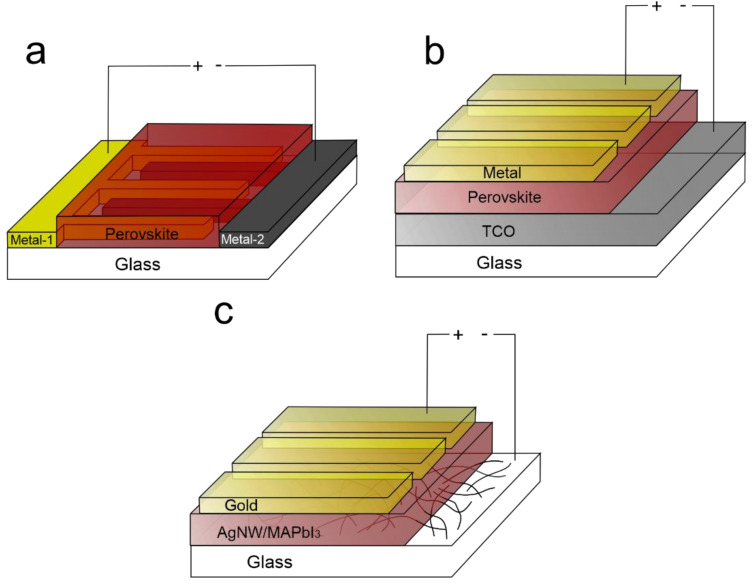
Schematic representation of MSM PPDs with different device designs; (**a**) lateral design; (**b**) vertical design; (**c**) hybrid design.

Generally, MSM structures, in which the work functions of the metallic layers are not similar ($\phi_{1}\;\neq \;\phi_{2}$; here, $\phi_{1}$ and $\phi_{2}$ refer to the work functions of the first and second metallic layers of PPDs), have two main device architectures: (1) vertical (also referred to as ‘sandwich’) and (2) lateral designs [47]. MSM PPDs with the vertical design represent devices in which the perovskite photo-absorber layer is sandwiched between two metallic electrodes (see Figure 1a). These devices are solution-processable and possess a faster response time due to smaller electrode spacing as opposed to devices with the lateral design [48]. However, in devices with the vertical design, one of the metallic electrodes must be transparent, which often limits the choice of materials used to manufacture MSM PPDs to the typical transparent conductive oxides (TCO) such as indium-doped or fluorine-doped tin oxides (ITO or FTO). Additionally, the light has to pass through this layer before reaching the perovskite photo-absorber layer. This leads to transmission losses due to reflection and absorption of the incident light by the substrate and the transparent metallic layer. Therefore, the lateral configuration (see Figure 1b) presents a better alternative in terms of light transmission losses.

The key issue with the lateral design for device architecture of MSM PPDs is that it requires setting the distance between the metallic contacts as close as possible for sufficient charge separation and transport. In the vertical designs, the distance between the metallic layers is set by the thickness of the perovskite layer. However, in PPDs with the lateral design, the minimum distance between the metallic layers is set by the limitations of the manufacturing method selected for the fabrication process. Usually, the metallic contacts in MSM PPDs with the lateral design are fabricated using lithography tools [49,50]. However, lithography processing is expensive and requires special operating conditions and facilities (cleanroom and nano- and micro-fabrication facilities). An advantageous combination of the vertical and lateral configurations could potentially resolve the key issues of both designs. A possible route is to embed one of the electrodes made of a network of metal nanowires or nanorods into the perovskite photo-absorber layer. Here, the embedded metal nanowire electrode can afford reduced transmission losses as opposed to TCOs that are often employed in MSM PPDs, both with vertical and lateral designs [51]. Additionally, a small distance between the individual metallic nanowires embedded in the perovskite layer can help to improve charge collection.

Herein, we demonstrate a self-powered MSM PPD (schematically represented in Figure 1c) in which one of the electrodes made of a network of silver nanowires (AgNW) is embedded in the perovskite layer, whereas a thin layer of gold, thermally evaporated directly onto the AgNW/perovskite film, serves as the other electrode of the MSM PPD. The obtained MSM PPDs have a hybrid design between the vertical and lateral designs. The manufacturing approach demonstrated here affords a facile and solution-processable method that can be an alternative for the lithography-based methods employed for manufacturing of MSM PPDs. The density of AgNWs can control the distance between the two metallic electrodes of MSM PPD, while the aspect ratio of AgNWs determine the work function of the embedded AgNW electrode. With such a device design, we have achieved a photoresponsivity of 2 × 10^−5^ AW^−1^ in the visible region and 4 × 10^−5^ AW^−1^ in the UV region. On average, the devices exhibit photocurrent values around 1.1 × 10^−6^ A. The ON/OFF ratio calculated from the photoresponse values of the devices operated under the illumination and in the dark conditions show a value of 83.4.

## 2. Materials and Methods

### 2.1. Materials

Methylammonium iodide (MAI, 99.995%) was purchased from GreatCellSolar. Lead(II) iodide (PbI_2_, anhydrous, 99%), acetone, 2-propanol (IPA), methylamine ethanol solution (33 wt%, MA/EtOH), acetonitrile (ACN), silver nitrate (AgNO_3_, 99.8%), polyvinylpyrrolidone (PVP, Mw = 360 k), sodium chloride (NaCl, 99.5%), potassium bromide (KBr, 99%), and ethylene glycol (EG, 99%) were purchased from Merck. Ethanol (C_2_H_5_OH, 99%) was purchased from a local supplier. All reagents were used as received without any further purification. 

### 2.2. Synthesis of Silver Nanowires

AgNWs were synthesized according to a protocol developed by Kaikanov et al. [52]. For the synthesis of AgNWs, 0.4 g of PVP was dissolved in 75 mL of EG at 130 °C in 30 min. Then, the EG solution of PVP waws allowed to cool down for the next 30 min on a hotplate at room temperature (RT). Afterwards, 0.5 g of AgNO_3_ powder was added to the solution and allowed to dissolve completely for 10 min at RT. All the aforementioned steps were performed while stirring the solution with a magnetic stirrer at a 500 rpm stirring rate. Next, 200 μL of NaCl with KBr solution in EG (NaCl with KBr solution in EG was prepared in advance in a separate vial: 0.1 g of NaCl and 0.015 g of KBr were dissolved in 20 mL of EG using an ultrasonic bath) was added to AgNO_3_ with PVP solution and stirred for two minutes at RT. Then, the mixture of AgNO_3_, PVP, NaCl, and KBr in EG was placed onto a hot plate at 130 °C and stirred at 500 rpm. After 10 min, the stirrer was turned off and the mixture was left to grow AgNWs at 130 °C for 5 h. Finally, the suspension was cooled down to RT. Acetone and ethanol were used to wash the precipitated AgNWs three times by centrifugation at 2000 rpm for 10 min. Finally, the obtained AgNWs were redispersed in ethanol.

### 2.3. Device Fabrication

**Figure 2:**
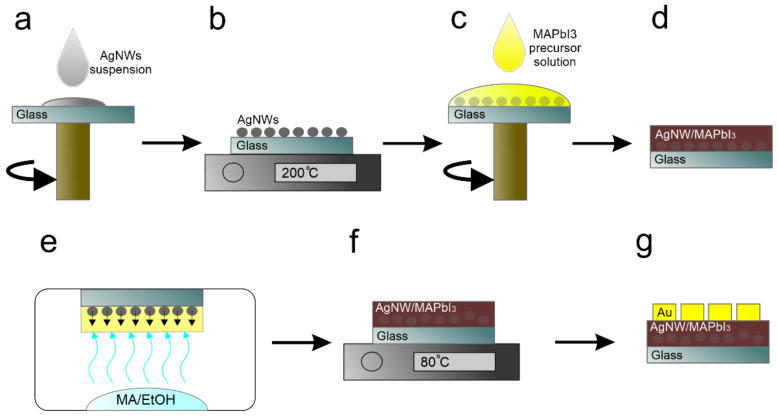
Schematic representation of fabrication of MSM PPDs. (**a**) Deposition of AgNW network layer on glass substrate; (**b**) Thermal annealing of AgNW network layer; (**c**) Deposition of MAPbI_3_ layer onto AgNW network layer; (**d**) Schematic cross-section of AgNW/MAPbI_3_ layer; (**e**) Liquefying of MAPbI_3_ layer by MA vapor treatment; (**f**) MAPbI_3_ layer with embedded AgNW network layer; (**g**) Schematic cross-section view of complete MSM PPDs.

A schematic diagram of fabrication of MSM PPDs with embedded AgNWs is depicted in Figure 2. For this, 2 × 2 cm^2^ soda-lime glass substrates are cleaned in an aqueous detergent, acetone, and IPA using an ultrasonic treatment for 15 min in each media. The cleaned substrates are treated with UV-Ozone for 25 min to enhance the wettability of the substrates. The obtained suspension of AgNWs in ethanol is used to prepare the AgNW network layers on glass substrates using a dynamic dispensed spin-coating method [53]. For this, 100 μL of AgNW suspension is dropped onto a clean and UV-Ozone-treated glass substrate while spinning the substrate at 1000 rpm (Figure 2a). This process is repeated until a desired conductivity of the AgNW network layer is achieved. Then, the samples are annealed at 200 °C for 20 s to remove the remaining PVP from the surface of AgNWs (Figure 2b). Next, the methylammonium lead triiodide (MAPbI_3_) perovskite photo-absorber layer is formed via spin casting (Figure 2c,d). For this, a perovskite precursor solution is spin-coated on glass substrates with the AgNW network layers at 1500 rpm for 30 s. The perovskite precursor solution is prepared by dissolving 0.159 g of MAI and 0.461 g of PbI_2_ in 0.6 mL of MA/EtOH. Then, 0.6 mL of ACN is dropped into the MAPbI_3_ precursor solution after 1 h of vigorous stirring at RT. Once the deposition of the MAPbI_3_ photo-absorber layer is complete, the samples are placed in a vessel filled with methylamine gas for 20 s (Figure 2e). Excess MA gas vapor dissolves the MAPbI_3_ layer, forming a liquefied solution in which MAI and PbI_2_ are dissolved in MA [54,55]. This step affords immersion of the AgNW network layer in the medium of the liquefied MAPbI_3_ layer. In the next step, the samples are placed on a hotplate at 80 °C for 1 min to crystalize the MAPbI_3_ of the AgNW/MAPbI_3_ layer (Figure 2f). Finally, the gold electrodes are deposited using the vacuum thermal evaporation method (Figure 2g), which completes the fabrication process of MSM PPD devices.

### 2.4. Characterization and Device Measurements

Surface morphology and topography of the samples were characterized using scanning electron microscopy (SEM, Zeiss Crossbeam 540, Oberkochen, Germany) and atomic force microscopy (AFM, AIST-NT SMART SPM 1000, Novato, CA, USA), respectively. Powder x-ray diffractograms (XRD) of the samples were obtained using the Rigaku Smartlab powder x-ray diffraction measurement system (Austin, TX, USA). An ultraviolet photoelectron spectrometer (UPS, NEXSA, Thermo Scientific, Paisley, UK) was used to determine the work function of AgNWs. Optical absorbance and conductivity of the samples were measured using a UV-Vis spectrometer (Lambda 1050, PerkinElmer, Waltham, MA, USA) and a four-point probing system (RM3000, Jandel, Leithon Buzzard, UK), respectively. The photoresponse measurements were carried out using a potentiostat (Autolab PGSTAT302N, Metrohm, Herisau, Switzerland) and white (cool) LED light (Metrohm). The spectral responsivity and detectivity measurements were performed using a solar simulator with a monochromator (TLS260–300X, Newport, Irvine, CA, USA)

## 3. Results

The obtained AgNWs are up to 50 µm in length and up to 100 nm in diameter (see Figure S1a,b). The XPS and UPS measurements are performed before and after thermal annealing of AgNW network layers on glass substrates at 200 °C to analyze the surface properties of the AgNWs and to estimate their work function values (see Figures S2 and S3, respectively, in Supplementary Materials). The comparison of the XPS spectra of as-deposited and annealed AgNW network layer shows that, after thermal annealing, the ratio of the Ag3d peak (368.9 eV) to C1s peak (286.1 eV) becomes ~30% larger, which indicates a reduced amount of PVP on the surface of the AgNWs (see Figure S2 in Supplementary Materials). The comparison of the UPS spectra of an as-deposited and annealed AgNW network layer indicates that the work function of the AgNW network layer changes slightly from its initial value of 3.21 eV before the annealing to 3.71 eV after the annealing (see Figure S3 in Supplementary Materials). A UPS spectrum of a neat silver film is shown in Figure S3 (see Supplementary Materials) as a reference.

**Figure 3:**
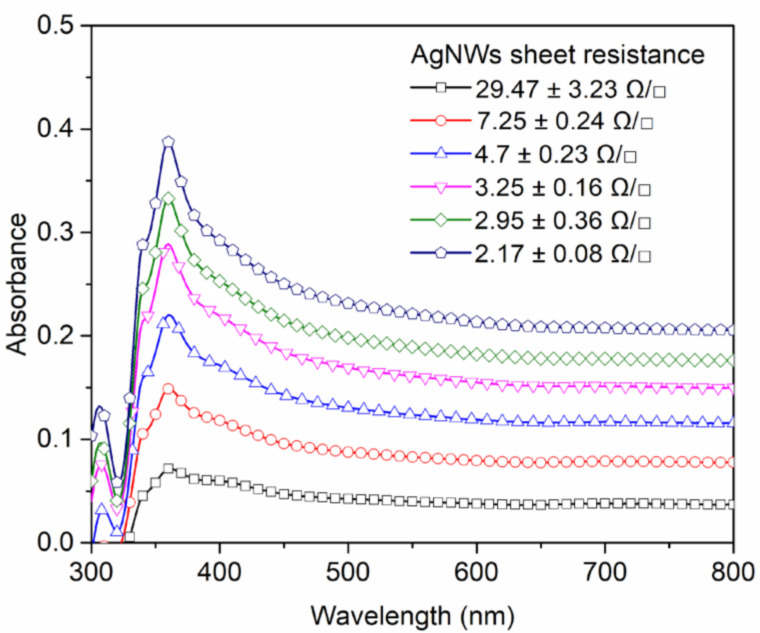
UV-Vis absorbance of AgNW network layers with different thickness and sheet resistance values.

In order to make MSM PPDs with the desired functionality, the AgNW network layer must have high electrical conductivity for acceptable charge-carrier transport and must be transparent enough for good light transmission. In our experiments, the thinnest AgNW network layers on glass substrates showed sheet resistance values around 30 Ω/□ (ohm per square) and maximum absorbance value of around 0.39 OD at ~360 nm, as shown in Figure 3. Increasing the thickness of the AgNW network layer results in films with higher absorbance and better electrical conductivity. It should be noted that, due to a large diameter of AgNWs (~100 nm), thicker AgNW network layers will have larger thickness and roughness. This might become an issue during device manufacturing, since a complete embedding of the AgNW network layer in the bulk of the perovskite layer might become very challenging. The analysis of the sample with AFM imaging (see Figure S4a in Supplementary Materials) shows that the AgNW network layers with sheet resistance values around 5 Ω/□ have thickness values around 600 nm. This is thin enough to form a good quality AgNW/MAPbI_3_ metal–semiconductor layer, in which the AgNW network layer is embedded in the bulk of the perovskite photo-absorber layer. Hence, fabrication of MSM PPDs is conducted using the AgNW network layers with sheet resistance values around 5 Ω/□.

**Figure 4:**
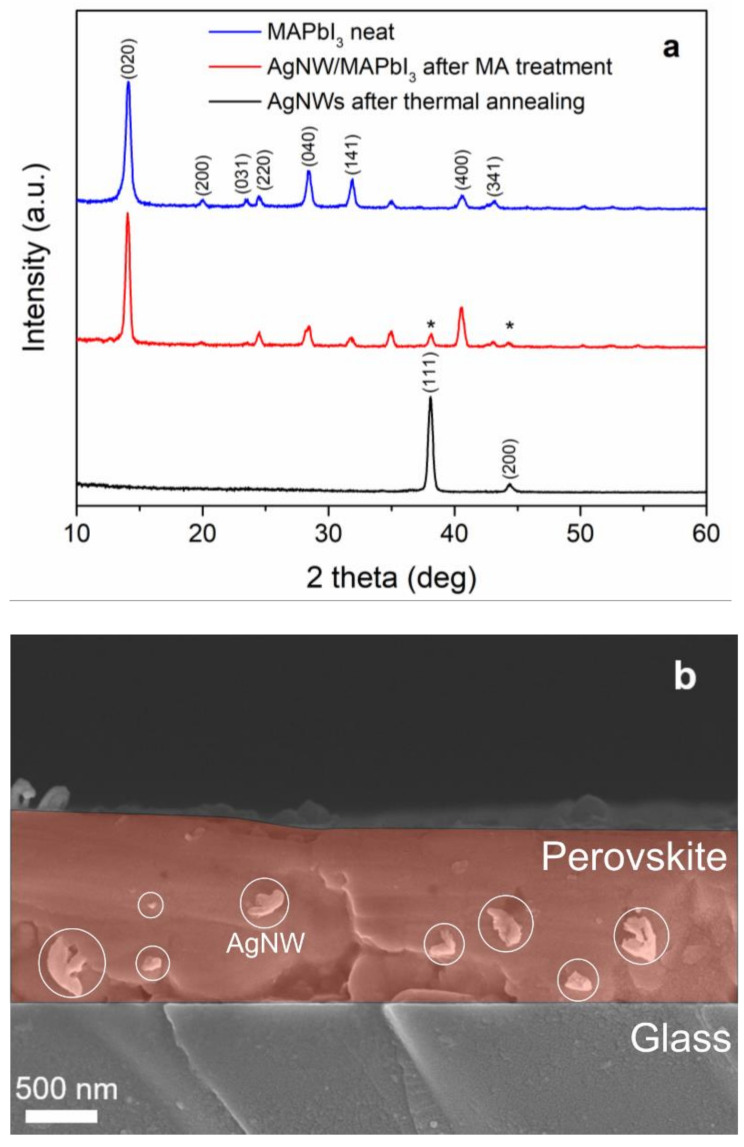
(**a**) XRD patterns of neat MAPbI_3_, AgNW/MAPbI_3_, and AgNW network layers. (**b**) Cross-sectional SEM image of AgNW/MAPbI_3_ layer. The star signs (*) in the AgNW/MAPbI_3_ XRD pattern indicate the reflections from (111) and (200) planes of silver nanowires.

The XRD measurements performed on the AgNW/MAPbI_3_ films indicate that the process of embedding does not affect the structural integrity of the AgNW network layer. X-ray diffraction pattern (see Figure 4a) of a AgNW/MAPbI_3_ film contains peaks originating both from the MAPbI_3_ (peaks at 14.08°, 20.02°, 23.4°, 24.5°, 28.36°, and 31.9°, assigned to (020), (200), (031), (220), (040), and (141) planes of orthorhombic neat MAPbI_3_) and the AgNW network layer (peaks at 38.08° and 44.36°, assigned to the reflections from the (111) and (200) planes of the face-centered cubic silver nanowires (JCPDS file No. 04–0783)) [56]. Figure 4b depicts a cross-sectional SEM image of a AgNW/MAPbI_3_ layer. The SEM image indicates that the AgNWs are somewhat embedded in the perovskite medium, which is evidenced by the AgNWs protruding from the cross-sectional surface of the AgNW/MAPbI_3_ layer (indicated by the circles in the image). The comparison of the optical micrographs taken from the glass substrate side of a AgNW/MAPbI_3_ layer before and after the MA gas treatment also indicates a partial embedding of the AgNWs in the perovskite layer medium (see Figure S5a,b in Supplementary Materials).

**Figure 5:**
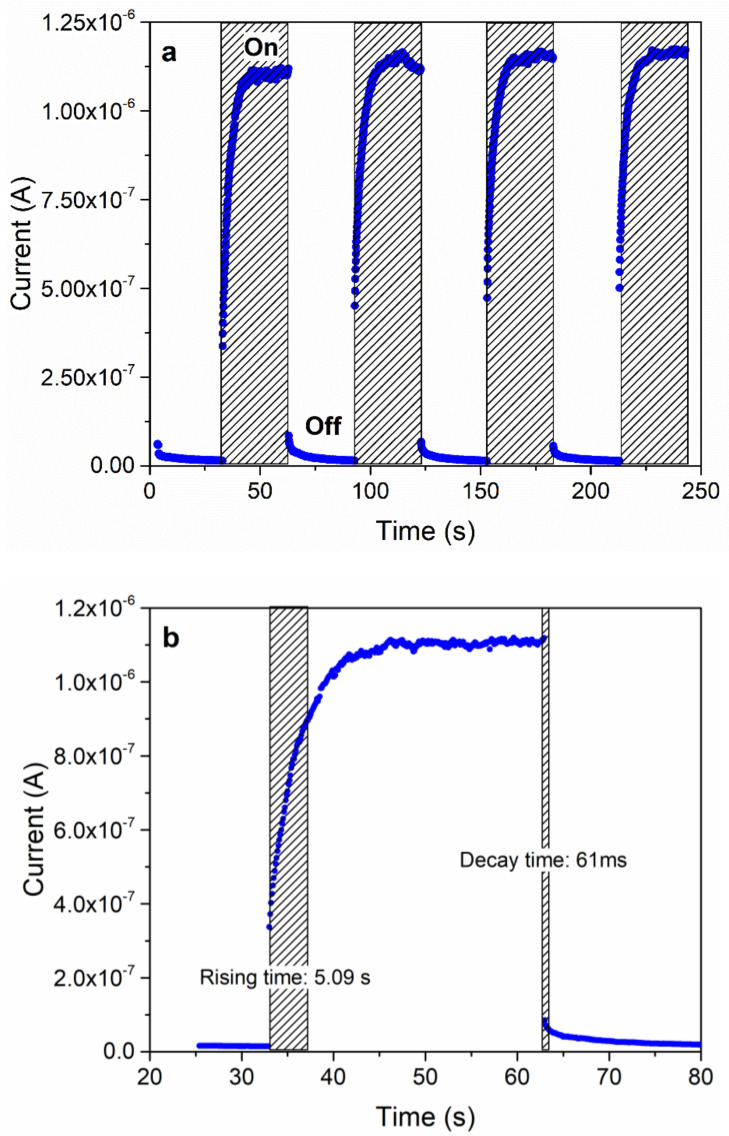
(**a**) *I–t* curve of MSM PPDs measured under zero applied bias and using a white LED light. (**b**) Estimation of the rise time and decay time of MSM PPDs from the *I–t* curve shown in (**a**).

Figure 5a shows a time-dependent photoresponse (*I–t*) curve of the obtained MSM PPDs with the embedded AgNW network electrodes recorded under a flashing white LED light illumination (illumination intensity is ~75 mW cm^−2^) at zero applied bias. The *I–t* curve clearly indicates that the obtained devices behave as PDs, showing two orders of magnitude increase in the device current (~1.1 × 10^−6^ A) under the illumination condition (device current in the dark is ~2 × 10^−8^ A). The active area of the device is 0.3 cm^2^ and the photoresponse measurements are performed without the use of a mask. The estimation of the response time (see Figure 5b) from the *I–t* curve indicates that the rise time and decay time of the obtained PPDs is around 5.09 s and 0.06 s, respectively. Figure S6a (Supplementary Materials) shows the current–voltage (*I–V*) curves of the MSM PPDs measured in the ambient atmosphere at RT in the dark and under different illumination conditions, using white LED light and neutral density filters. The *I–V* curves exhibit a photodiode behavior in which the device current increases more or less linearly with the illumination intensity. A similar trend is also observed in the *I–t* curve of the MSM PPDs measured under zero applied bias and flashing white LED light with varying intensity (see Figure S6b in Supplementary Materials). A plot of the photoresponse vs. illumination intensity obtained from the analysis of Figure S6b also shows that the device current is somewhat linearly proportional to the illumination intensity (see Figure S6c in Supplementary Materials). The photoresponse measurements under applied bias ranging from −2 V to 2 V indicate that, with applied bias, the photoresponse of the MSM PPDs can be increased (see Figure S7 in Supplementary Materials).

Responsivity (R) and detectivity D (*) are some of the important parameters for PDs. Responsivity of PDs is calculated from the following relation [45]:

(1) $$R=\frac{I_{P}}{PS}$$

where photocurrent is the difference between device currents under illumination and in the dark ($I_{P}=I_{light}-I_{dark}$). Here, $I_{light}$ and $I_{dark}$ are device currents under illumination and dark conditions, correspondingly. P is the incident light intensity, and S is the effective device area.

Detectivity of PDs is calculated from the following relation [57,58]:

(2) $$D^{*}=\frac{R}{\sqrt{\frac{4k_{B}T}{R^{\prime }S}+\frac{2qI_{dark}}{S}}},$$

where R is responsivity [A/W], $I_{dark}$ is dark current, $k_{B}$ is the Boltzmann constant, T is a temperature, $R^{\prime }$ is a series resistance, and q is the elementary charge.

**Figure 6:**
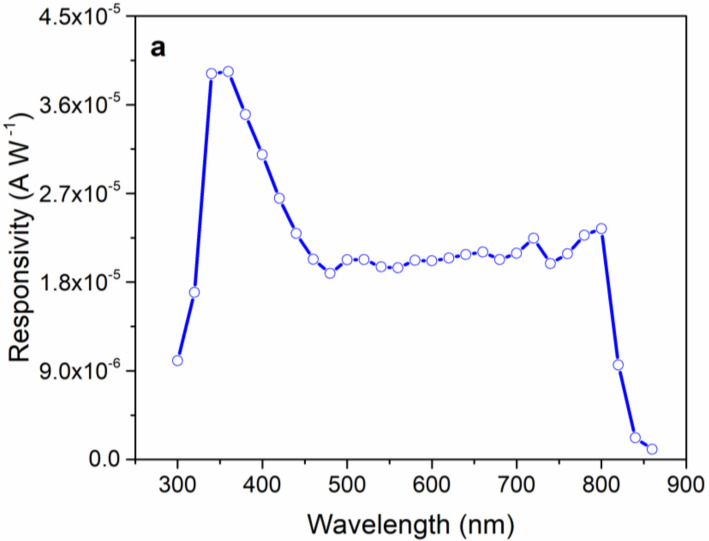

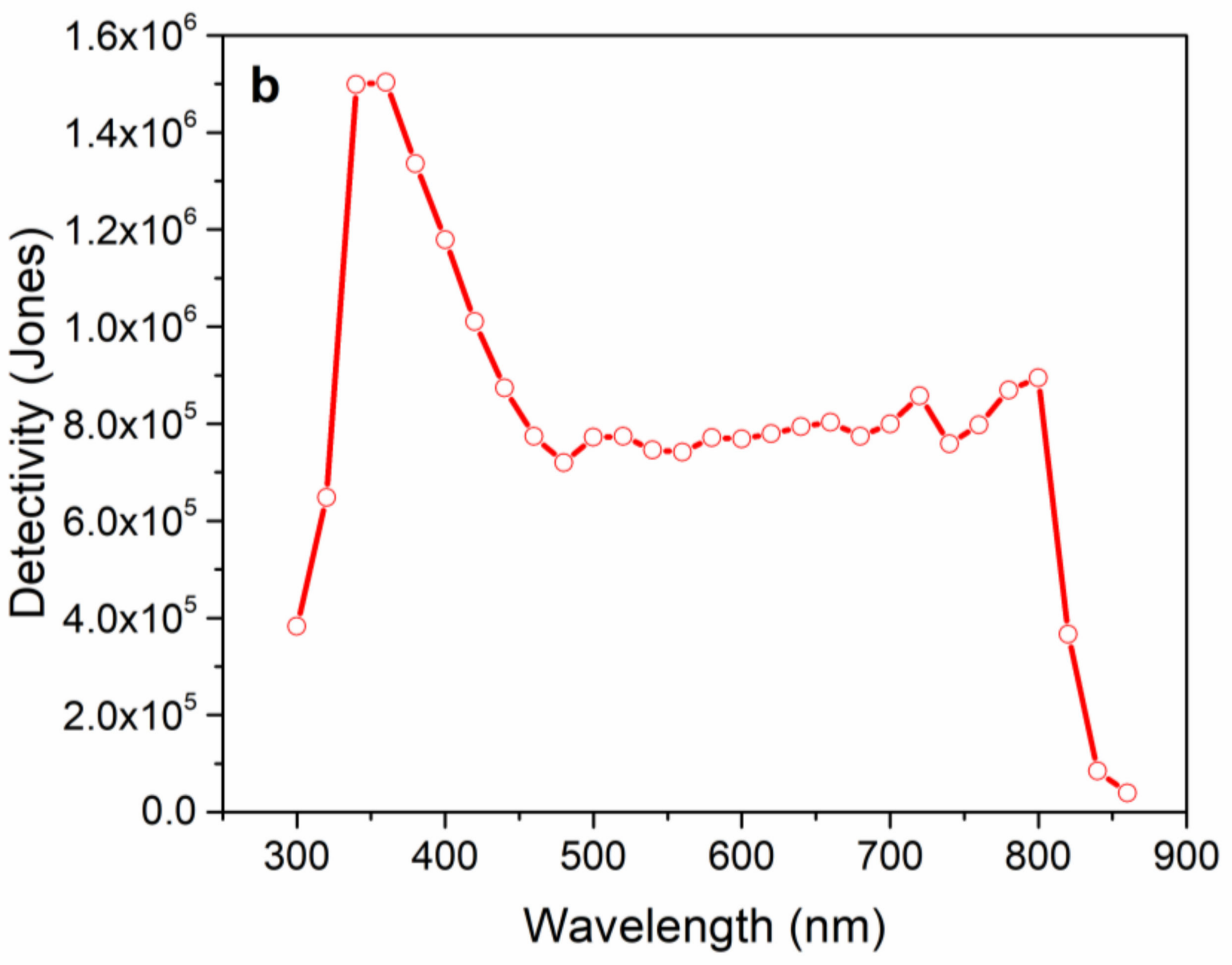
Spectral dependence of responsivity (**a**) and detectivity (**b**) of MSM PPDs.

The spectral dependence of responsivity ($R\left(\lambda \right)$) is estimated by using Equation (1) and using the device current values measured in the dark and under illumination with monochromatic light at different wavelengths. Figure 6a shows the device $R\left(\lambda \right)$ values for the wavelength range of 300 to 870 nm. The $R\left(\lambda \right)$ spectrum indicates that the take-off value of responsivity is at ~850 nm, which is around the absorption edge of the MAPbI_3_ perovskite photo-absorber layer (see Figure S8 in Supplementary Materials). From ~800 to ~450 nm, $R\left(\lambda \right)$ levels off, showing responsivity values around 2 × 10^−5^ AW^−1^. Starting from ~450 nm, $R\left(\lambda \right)$ increases further, peaking at 350 nm with a responsivity value of around 4 × 10^−5^ AW^−1^. It is noteworthy to mention that the behavior of the $R\left(\lambda \right)$ spectrum between 350 and 450 nm is somewhat consistent with the absorption profile of AgNWs (see Figure 3). This suggests that the enhancement of the responsivity values in this spectral region, perhaps, may originate from the surface plasmon effects associated with AgNWs, which enhance light absorption by the MAPbI_3_ layer at the AgNW/MAPbI_3_ interface [59,60]. Below 350 nm, $R\left(\lambda \right)$ rapidly decreases, showing a responsivity value of ~0.9 × 10^−5^ AW^−1^ at 300 nm. The decrease in the responsivity values below 350 nm is due to blocking (absorption) of the incident light by the substrate (soda-lime glass), as shown in Figure S9 (Supplementary Materials).

Figure 6b shows the spectral dependence of detectivity ($D^{*}\left(\lambda \right)$) of the devices estimated using Equation (2). The $D^{*}\left(\lambda \right)$ spectrum shows a similar trend as in $R\left(\lambda \right)$ due to direct proportionality of R and D (see Equation (2)). Here, the take-off value of the $D^{*}\left(\lambda \right)$ is at ~850 nm, and it levels off between the ~450 and 800 nm spectral range at the values around 8 ×10^5^ Jones. Similar to $R\left(\lambda \right)$, $D^{*}\left(\lambda \right)$ increases further from 450 to 350 nm, showing a maximum detectivity value of ~1.5 × 10^6^ Jones at 350 nm. Below 350 nm, detectivity of the devices quickly decreases, showing 4 × 10^5^ Jones at 300 nm. The decrease in detectivity below 350 nm is again associated with blocking of the incident light by the substrate (see Figure S9 in Supplementary Materials). Overall, the spectral dependences of responsivity and detectivity presented in Figure 6 show that the devices can also operate in the UV region (between 350 and 450 nm), which is an additional advantage of the MSM PPDs presented in this work over other MSM PPDs that employ typical TCO layers as one of the transparent metallic contacts [61,62].

## 4. Discussion

The SEM images shown in Figure S1 (Supplementary Materials) reveal that the distribution of the AgNWs is not homogeneous and the ‘holes’ (transparent or empty areas) in the AgNW network are generally large, reaching several µm^2^ in size. While the AgNW network layers with the optimized sheet resistance and transmittance values are used to manufacture MSM PPDs, it should be noted that the diffusion length of charge carries in typical solution-processed polycrystalline MAPbI_3_ films are in the submicron range. Hence, the large ‘holes’ in the AgNW network layer can hinder effective charge collection in the devices since the majority of the photogenerated charge carries recombine before reaching the respective metallic electrodes. One way to resolve this issue would be to use AgNWs with smaller diameter. In this way, the AgNW network layer can be made denser, while keeping its transmittance low. Furthermore, the UPS measurements (see Figure S3 in Supplementary Materials) indicate that the work function of the AgNW network layer is around −3.71 eV, which is slightly higher than the conduction band edge of MAPbI_3_ (see Figure S10 in Supplementary Materials). To address this issue, tuning of the AgNW aspect ratio or use of surface-modification strategies (e.g., using self-assembled monolayers to tune the work function) would be an alternative way to achieve a desired work function value [63,64,65]. In this regard, the use of metallic nanowires made of other materials such as gold would improve the stability of devices, due to the more inert nature of gold and its better chemical stability to perovskites [66]. Additionally, it is challenging to achieve full immersion of the AgNW network layer in the perovskite medium. Some fraction of the AgNW network layer remains between the glass substrate and perovskite layer, causing certain transmission losses, whereas too-rough a AgNW network layer may cause shunting in devices. These issues could be the reasons for the moderate performances of the devices presented in this work. Nevertheless, the presented approach demonstrates a facile and solution-processable manufacturing of self-powered and broadband MSM PPDs that employ nanoscale materials. The presented MSM PPDs have a unique device structure and can be a good alternative for fabricating cost-effective PDs without the use of expensive lithography tools.

## 5. Conclusions

In conclusion, a manufacturing of self-powered MSM PPDs with embedded AgNW network electrodes has been demonstrated. On average, the devices exhibit ~1.1 × 10^−6^ A device current under LED white light illumination (75 mW cm^−2^). Responsivity of the devices in the visible region is around 2 × 10^−5^ AW^−1^, while in the UV region it is a factor of two higher (4 × 10^−5^ AW^−1^). The presented approach is fully solution-processable and serves as a cost-effective alternative for manufacturing of broadband MSM PDs with perovskites or other solution-processed semiconductor materials.

## Data Availability

The data presented in this study are available on request from the corresponding author.

## Supplementary Materials

The following supporting information can be downloaded at: https://www.mdpi.com/article/10.3390/nano12071034/s1, Figure S1: Scanning electron microscope (SEM) images of AgNW network layer on glass substrates. (a) Low-magnification (×500) SEM image of AgNW network layer. (b) High-magnification (×10,000) SEM image of AgNW network layer; Figure S2: X-ray photoelectron spectra of AgNW network layer. (a) As-prepared AgNW network layer. (b) AgNW network after thermal annealing at 200 °C for 20 s.; Figure S3: Ultraviolet photoelectron spectroscopy (UPS) analysis of samples. (a) UPS spectra of silver film, as-prepared AgNW network layer (before thermal annealing), and AgNW network layer after thermal annealing at 200 °C for 20 s. (b) The edges of the UPS spectra with the indicated Fermi levels. (c) Low-energy cutoffs of the UPS spectra with highlighted onsets for determination of work functions; Figure S4: Atomic force microscopy (AFM) imaging of samples. (a) AFM image of AgNW network layer on glass substrate. (b) AFM image of AgNW/MAPbI_3_ layer; Figure S5. Optical microscope image of AgNW/MAPbI3 layer from the backside of the glass substrate (a) before MA treatment; (b) after MA treatment; Figure S6: (a) *I–V* curves of MSM PPDs with embedded AgNW network electrodes. (b) *I–t* curve of MSM PPDs with embedded AgNW network electrodes, measured under zero bias and flashing white LED light with varying intensity. (c) Photocurrent vs. intensity plot obtained from the analysis of the *I–t* curve shown in (b); Figure S7: *I–t* curve of MSM PPDs with the embedded AgNW network electrode, measured under different applied bias and flashing white LED light with a constant intensity (75 mW cm^−2^); Figure S8: (a) UV-Vis absorbance spectra of MAPbI_3_ and AgNW/MAPbI3 films; (b) Tauc plot originated from the absorption spectra of neat MAPbI_3_; Figure S9: UV-Vis transmittance spectrum of soda-lime glass substrate; Figure S10. Band diagram for the device shown in Figure 1c.

## References

[1] Li L., Ye S., Qu J., Zhou F., Song J., Shen G. (2021). Recent Advances in Perovskite Photodetectors for Image Sensing. Small.

[2] Li C., Ma Y., Xiao Y., Shen L., Ding L. (2020). Advances in perovskite photodetectors. InfoMat.

[3] Thrush E., Levi O., Ha W., Carey G., Cook L., Deich J., Smith S., Moerner W., Harris J. (2004). Integrated semiconductor vertical-cavity surface-emitting lasers and PIN photodetectors for biomedical fluorescence sensing. IEEE J. Quantum Electron..

[4] Huang B., Liu J., Han Z., Gu Y., Yu D., Xu X., Zou Y. (2020). High-Performance Perovskite Dual-Band Photodetectors for Potential Applications in Visible Light Communication. ACS Appl. Mater. Interfaces.

[5] Shi Q., Dong B., He T., Sun Z., Zhu J., Zhang Z., Lee C. (2020). Progress in wearable electronics/photonics—Moving toward the era of artificial intelligence and internet of things. InfoMat.

[6] Cai S., Xu X., Yang W., Chen J., Fang X. (2019). Materials and Designs for Wearable Photodetectors. Adv. Mater..

[7] Chen H., Liu H., Zhang Z., Hu K., Fang X. (2015). Nanostructured Photodetectors: From Ultraviolet to Terahertz. Adv. Mater..

[8] Mukhokosi E.P., Manohar G.V., Nagao T., Krupanidhi S.B., Nanda K.K. (2020). Device Architecture for Visible and Near-Infrared Photodetectors Based on Two-Dimensional SnSe_2_ and MoS_2_: A Review. Micromachines.

[9] Huo N., Konstantatos G. (2018). Recent Progress and Future Prospects of 2D-Based Photodetectors. Adv. Mater..

[10] Geng D., Yang H.Y. (2018). Recent Advances in Growth of Novel 2D Materials: Beyond Graphene and Transition Metal Dichalcogenides. Adv. Mater..

[11] Dong C., Wang Z.K., Liao L.S. (2020). Progress of Triple Cation Organometal Halide Perovskite Solar Cells. Energy Technol..

[12] Xie C., Yan F. (2017). Flexible Photodetectors Based on Novel Functional Materials. Small.

[13] Teng F., Hu K., Ouyang W., Fang X. (2018). Photoelectric Detectors Based on Inorganic p-Type Semiconductor Materials. Adv. Mater..

[14] Zheng X., Bai Y., Xiao S., Meng X., Zhang T., Yang S. (2017). Strategies for Improving Efficiency and Stability of Perovskite Solar Cells. MRS Adv..

[15] Peng J., Chen Y., Zheng K., Pullerits T., Liang Z. (2017). Insights into charge carrier dynamics in organo-metal halide perovskites: From neat films to solar cells. Chem. Soc. Rev..

[16] Adinolfi V., Peng W., Walters G., Bakr O.M., Sargent E.H. (2017). The Electrical and Optical Properties of Organometal Halide Perovskites Relevant to Optoelectronic Performance. Adv. Mater..

[17] Guner T., Demir M.M. (2018). A Review on Halide Perovskites as Color Conversion Layers in White Light Emitting Diode Applications. Phys. Status Solidi.

[18] Shi Z., Jayatissa A. (2018). Perovskites-Based Solar Cells: A Review of Recent Progress, Materials and Processing Methods. Materials.

[19] Wang Y., Yang D., Zhou X., Ma D., Vadim A., Ahamad T., Alshehri S.M. (2017). Perovskite/Polymer Hybrid Thin Films for High External Quantum Efficiency Photodetectors with Wide Spectral Response from Visible to Near-Infrared Wavelengths. Adv. Opt. Mater..

[20] Kumawat N.K., Dey A., Narasimhan K.L., Kabra D. (2015). Near Infrared to Visible Electroluminescent Diodes Based on Organometallic Halide Perovskites: Structural and Optical Investigation. ACS Photonics.

[21] Dong Q., Lei L., Mendes J., So F. (2020). Operational stability of perovskite light emitting diodes. J. Phys. Mater..

[22] Du P., Gao L., Tang J. (2020). Focus on performance of perovskite light-emitting diodes. Front. Optoelectron..

[23] Li Z., Moon J., Gharajeh A., Haroldson R., Hawkins R., Hu W., Zakhidov A., Gu Q. (2018). Room-Temperature Continuous-Wave Operation of Organometal Halide Perovskite Lasers. ACS Nano.

[24] Wang Y.C., Li H., Hong Y.H., Hong K.B., Chen F.C., Hsu C.H., Lee R.K., Conti C., Kao T.S., Lu T.C. (2019). Flexible Organometal–Halide Perovskite Lasers for Speckle Reduction in Imaging Projection. ACS Nano.

[25] Ippili S., Jella V., Eom S., Hong S., Yoon S.G. (2020). Light-Driven Piezo- and Triboelectricity in Organic—Inorganic Metal Trihalide Perovskite toward Mechanical Energy Harvesting and Self-powered Sensor Application. ACS Appl. Mater. Interfaces.

[26] Wu H., Ge Y., Niu G., Tang J. (2021). Metal Halide Perovskites for X-Ray Detection and Imaging. Matter.

[27] Yu X., Tsao H.N., Zhang Z., Gao P. (2020). Miscellaneous and Perspicacious: Hybrid Halide Perovskite Materials Based Photodetectors and Sensors. Adv. Opt. Mater..

[28] Perumal Veeramalai C., Feng S., Zhang X., Pammi S.V.N., Pecunia V., Li C. (2021). Lead–Halide perovskites for next-generation self-powered photodetectors: A comprehensive review. Photonics Res..

[29] Ding J., Du S., Zuo Z., Zhao Y., Cui H., Zhan X. (2017). High Detectivity and Rapid Response in Perovskite CsPbBr_3_ Single-Crystal Photodetector. J. Phys. Chem. C.

[30] Ding J., Fang H., Lian Z., Li J., Lv Q., Wang L., Sun J.L., Yan Q. (2016). A self-powered photodetector based on a CH_3_NH_3_PbI_3_ single crystal with asymmetric electrodes. CrystEngComm.

[31] Zhang Z., Chen K., Xia W., Zuo Z. (2020). MAPbBr_3_ single crystal based metal-semiconductor-metal photodetector enhanced by localized surface plasmon. Mater. Res. Express.

[32] Cai W., Li H., Li M., Wang M., Wang H., Chen J., Zang Z. (2021). Opportunities and challenges of inorganic perovskites in high-performance photodetectors. J. Phys. D Appl. Phys..

[33] Wang Y., Chang S., Chen X., Ren Y., Shi L., Liu Y., Zhong H. (2019). Rapid Growth of Halide Perovskite Single Crystals: From Methods to Optimization Control. Chin. J. Chem..

[34] Ahmadi M., Wu T., Hu B. (2017). A Review on Organic–Inorganic Halide Perovskite Photodetectors: Device Engineering and Fundamental Physics. Adv. Mater..

[35] Li Z., Li J., Ding D., Yao H., Liu L., Gong X., Tian B., Li H., Su C., Shi Y. (2018). Direct Observation of Perovskite Photodetector Performance Enhancement by Atomically Thin Interface Engineering. ACS Appl. Mater. Interfaces.

[36] Shrestha S., Tsai H., Yoho M., Ghosh D., Liu F., Lei Y., Tisdale J., Baldwin J., Xu S., Neukirch A.J. (2020). Role of the Metal-Semiconductor Interface in Halide Perovskite Devices for Radiation Photon Counting. ACS Appl. Mater. Interfaces.

[37] Bie Y.Q., Liao Z.M., Zhang H.Z., Li G.R., Ye Y., Zhou Y.B., Xu J., Qin Z.X., Dai L., Yu D.P. (2011). Self-Powered, ultrafast, visible-blind UV detection and optical logical operation based on ZnO/GaN nanoscale p-n junctions. Adv. Mater..

[38] Mohan D.G., Gopi S., Rajasekar V., Krishnan K., Mohan D.G., Gopi S., Selvarajan L., Rajavel R., Prakash B., Mohan D.G. (2019). High-Gain and fast-response metal-semiconductor-metal structured organolead halide perovskite photodetectors. Mater. Today Proc..

[39] Dong R., Fang Y., Chae J., Dai J., Xiao Z., Dong Q., Yuan Y., Centrone A., Zeng X.C., Huang J. (2015). High-Gain and Low-Driving-Voltage Photodetectors Based on Organolead Triiodide Perovskites. Adv. Mater..

[40] Dou L., Yang Y.M., You J., Hong Z., Chang W.H., Li G., Yang Y. (2014). Solution-Processed hybrid perovskite photodetectors with high detectivity. Nat. Commun..

[41] Fang Y., Huang J. (2015). Resolving weak light of sub-picowatt per square centimeter by hybrid perovskite photodetectors enabled by noise reduction. Adv. Mater..

[42] An Y., Behnam A., Pop E., Ural A. (2013). Metal-Semiconductor-Metal photodetectors based on graphene/p-type silicon Schottky junctions. Appl. Phys. Lett..

[43] Chen L.C., Lee K.L., Lee K.Y., Huang Y.W., Lin R.M. (2020). Study of Metal–Semiconductor–Metal CH_3_NH_3_PbBr_3_ Perovskite Photodetectors Prepared by Inverse Temperature Crystallization Method. Sensors.

[44] Bhatt V., Kumar M., Yadav P., Kumar M., Yun J.H. (2018). Low cost and solution processible sandwiched CH_3_NH_3_PbI_3-x_Cl_x_ based photodetector. Mater. Res. Bull..

[45] Perumal Veeramalai C., Yang S., Zhi R., Sulaman M., Saleem M.I., Cui Y., Tang Y., Jiang Y., Tang L., Zou B. (2020). Solution-Processed, Self-Powered Broadband CH_3_NH_3_PbI_3_ Photodetectors Driven by Asymmetric Electrodes. Adv. Opt. Mater..

[46] Meng J., Li Z. (2020). Schottky-Contacted Nanowire Sensors. Adv. Mater..

[47] Miao J., Zhang F. (2019). Recent progress on highly sensitive perovskite photodetectors. J. Mater. Chem. C.

[48] Hao D., Zou J., Huang J. (2019). Recent developments in flexible photodetectors based on metal halide perovskite. InfoMat.

[49] Wang H., Haroldson R., Balachandran B., Zakhidov A., Sohal S., Chan J.Y., Zakhidov A., Hu W. (2016). Nanoimprinted Perovskite Nanograting Photodetector with Improved Efficiency. ACS Nano.

[50] Georgiadou D.G., Lin Y., Lim J., Ratnasingham S., McLachlan M.A., Snaith H.J., Anthopoulos T.D. (2019). High Responsivity and Response Speed Single-Layer Mixed-Cation Lead Mixed-Halide Perovskite Photodetectors Based on Nanogap Electrodes Manufactured on Large-Area Rigid and Flexible Substrates. Adv. Funct. Mater..

[51] Jin W.Y., Ginting R.T., Ko K.J., Kang J.W. (2016). Ultra-Smooth, Fully Solution-Processed Large-Area Transparent Conducting Electrodes for Organic Devices. Sci. Rep..

[52] Kaikanov M., Kemelbay A., Amanzhulov B., Demeuova G., Akhtanova G., Bozheyev F., Tikhonov A. (2021). Electrical conductivity enhancement of transparent silver nanowire films on temperature-sensitive flexible substrates using intense pulsed ion beam. Nanotechnology.

[53] Ma Y., Vashishtha P., Chen K., Peach E.L., Ohayon D., Hodgkiss J.M., Halpert J.E. (2017). Controlled Growth of CH_3_NH_3_PbI_3_ Using a Dynamically Dispensed Spin-Coating Method: Improving Efficiency with a Reproducible PbI_2_ Blocking Layer. ChemSusChem.

[54] Yang J.A., Qin T., Xie L., Liao K., Li T., Hao F. (2019). Methylamine-Induced defect-healing and cationic substitution: A new method for low-defect perovskite thin films and solar cells. J. Mater. Chem. C.

[55] Zhang Y., Grancini G., Fei Z., Shirzadi E., Liu X., Oveisi E., Tirani F.F., Scopelliti R., Feng Y., Nazeeruddin M.K. (2019). Auto-Passivation of crystal defects in hybrid imidazolium/methylammonium lead iodide films by fumigation with methylamine affords high efficiency perovskite solar cells. Nano Energy.

[56] Shi Y.E., Li L., Yang M., Jiang X., Zhao Q., Zhan J. (2014). A disordered silver nanowires membrane for extraction and surface-enhanced Raman spectroscopy detection. Analyst.

[57] Chen Y., Wang Y., Wang Z., Gu Y., Ye Y., Chai X., Ye J., Chen Y., Xie R., Zhou Y. (2021). Unipolar barrier photodetectors based on van der Waals heterostructures. Nat. Electron..

[58] Wang J., Fang H., Wang X., Chen X., Lu W., Hu W. (2017). Recent progress on localized field enhanced two-dimensional material photodetectors from ultraviolet—Visible to infrared. Small.

[59] Pasupuleti K.S., Reddeppa M., Park B.G., Peta K.R., Oh J.E., Kim S.G., Kim M.D. (2020). Ag Nanowire-Plasmonic-Assisted Charge Separation in Hybrid Heterojunctions of Ppy-PEDOT:PSS/GaN Nanorods for Enhanced UV Photodetection. ACS Appl. Mater. Interfaces.

[60] Wang H., Lim J.W., Quan L.N., Chung K., Jang Y.J., Ma Y., Kim D.H. (2018). Perovskite-Gold Nanorod Hybrid Photodetector with High Responsivity and Low Driving Voltage. Adv. Opt. Mater..

[61] Shi L., Chen K., Zhai A., Li G., Fan M., Hao Y., Zhu F., Zhang H., Cui Y. (2020). Status and Outlook of Metal–Inorganic Semiconductor–Metal Photodetectors. Laser Photonics Rev..

[62] Dong Y., Zou Y., Song J., Song X., Zeng H. (2017). Recent progress of metal halide perovskite photodetectors. J. Mater. Chem. C.

[63] Reineck P., Brick D., Mulvaney P., Bach U. (2016). Plasmonic Hot Electron Solar Cells: The Effect of Nanoparticle Size on Quantum Efficiency. J. Phys. Chem. Lett..

[64] De Boer B., Hadipour A., Mandoc M.M., van Woudenbergh T., Blom P.W.M. (2005). Tuning of Metal Work Functions with Self-Assembled Monolayers. Adv. Mater..

[65] Lin X., Jumabekov A.N., Lal N.N., Pascoe A.R., Gómez D.E., Duffy N.W., Chesman A.S.R., Sears K., Fournier M., Zhang Y. (2017). Dipole-Field-Assisted charge extraction in metal-perovskite-metal back-contact solar cells. Nat. Commun..

[66] Li C., Tscheuschner S., Paulus F., Hopkinson P.E., Kießling J., Köhler A., Vaynzof Y., Huettner S. (2016). Iodine Migration and its Effect on Hysteresis in Perovskite Solar Cells. Adv. Mater..

