# Supplementary material for: Self-Powered Organometal Halide Perovskite Photodetector with Embedded Silver Nanowires
Source: Nanomaterials (Basel). 2022 Mar 22;12(7):1034. doi: 10.3390/nano12071034 (PMC9000456; doi:10.3390/nano12071034)
Supplement: Supplementary file 1 [file nanomaterials-12-01034-s001.zip › nanomaterials-1608241-supplementary.pdf]

Supplementary Materials

Self-Powered Organometal Halide Perovskite Photodetector with Embedded Silver Nanowires

Almaz R. Beisenbayev [1,†], Zhandos T. Sadirkhanov [2,†], Yerassyl Yerlanuly [2], Marat I. Kaikanov [2] and Askhat N. Jumabekov [2,*]

[1] Department of Chemical Engineering, Nazarbayev University, Nur-Sultan 010000, Kazakhstan; almaz.beisenbayev@nu.edu.kz (A.R.B)
[2] Department of Physics, Nazarbayev University, Nur-Sultan 010000, Kazakhstan; zhandos.sadirkhanov@nu.edu.kz (Z.T.S.); yerassyl.yerlanuly@nu.edu.kz (Y.Y.); marat.kaikanov@nu.edu.kz (M.I.K.)
* Correspondence: askhat.jumabekov@nu.edu.kz
† These authors contributed equally.

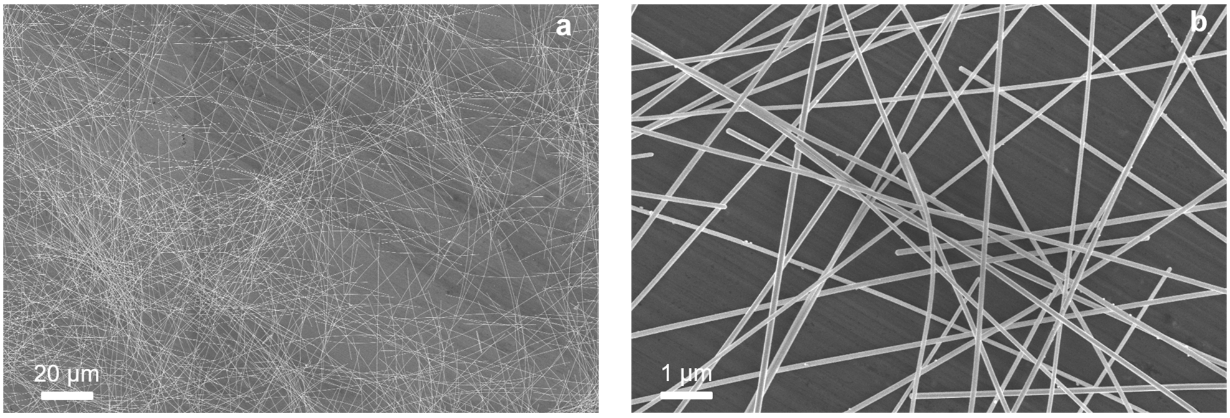

Figure S1. Scanning electron microscope (SEM) images of AgNW network layer on glass substrates. a) Low-magnification (×500) SEM image of AgNW network layer. b) High-magnification (×10000) SEM image of AgNW network layer.

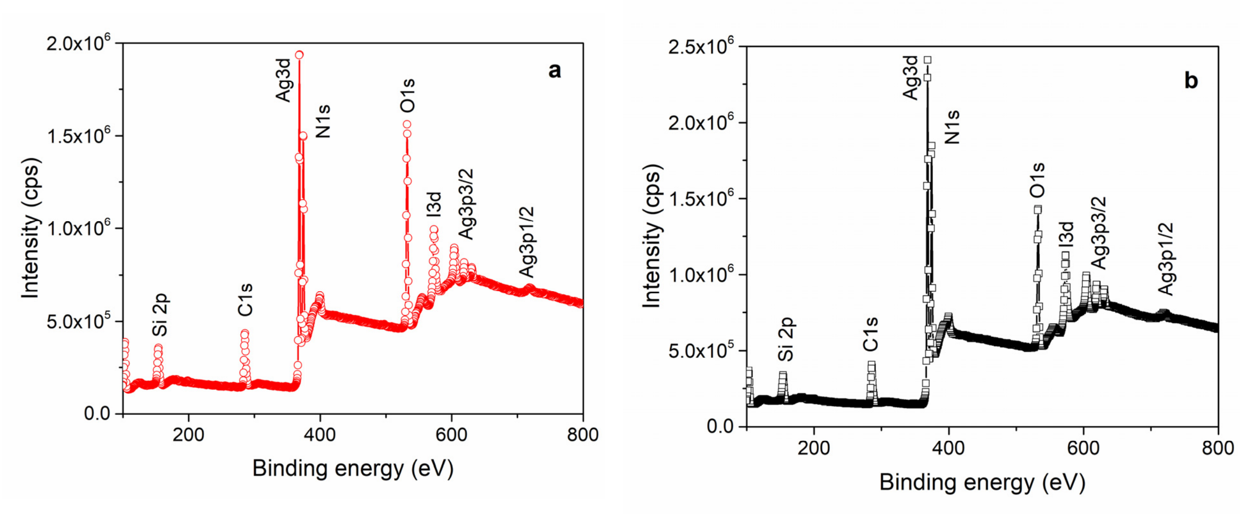

Figure S2. X-ray photoelectron spectra of AgNW network layer. a) As prepared AgNW network layer. b) AgNW network after thermal annealing at 200 °C for 20 sec.

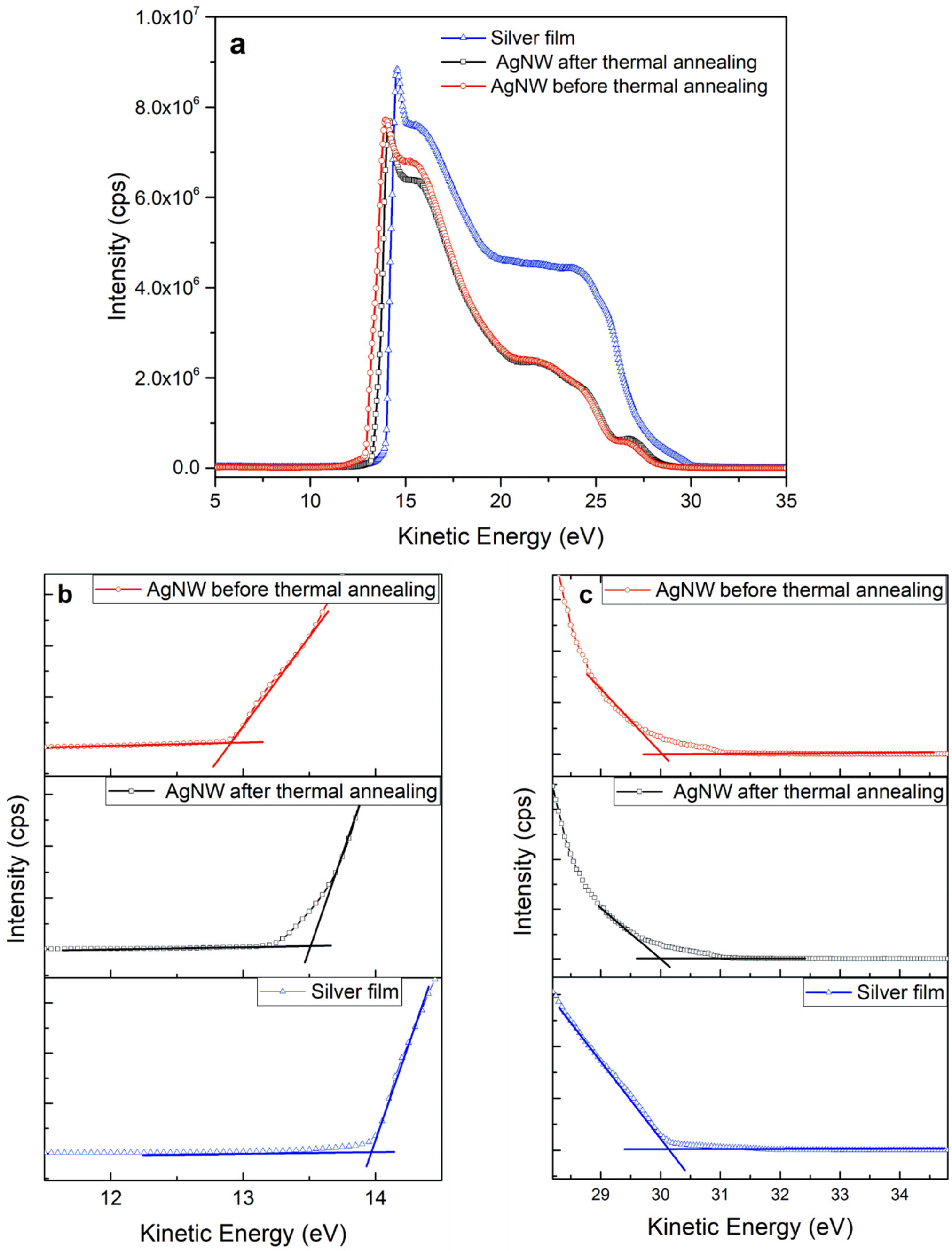

Figure S3. Ultraviolet photoelectron spectroscopy (UPS) analysis of samples. a) UPS spectra of silver film, as prepared AgNW network layer (before thermal annealing), and AgNW network layer after thermal annealing at 200 °C for 20 sec. b) The edges of the UPS spectra with the indicated Fermi levels. c) Low energy cutoffs of the UPS spectra with highlighted the onsets for determination of work functions.

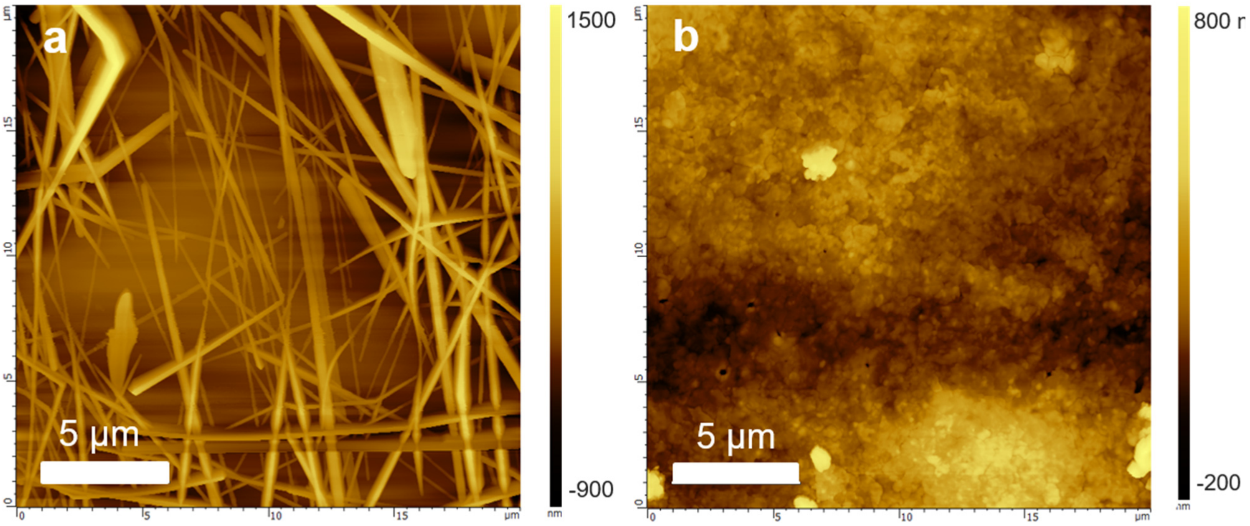

Figure S4. Atomic force microscopy (AFM) imaging of samples. a) AFM image of AgNW network layer on glass substrate. b) AFM image of AgNW/$MAPbI_3$ layer.

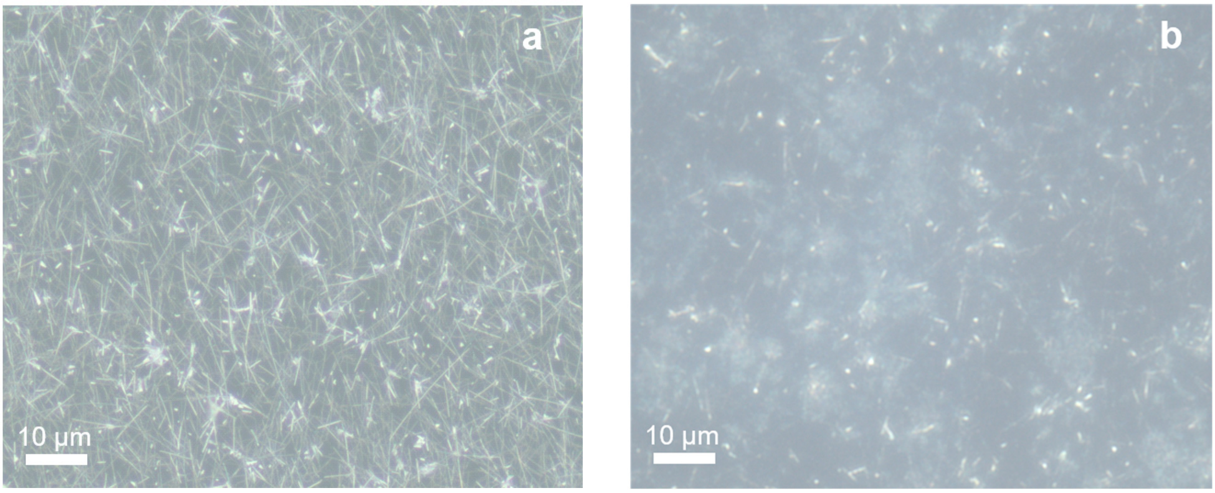

Figure S5. Optical micrographs of AgNW/$MAPbI_3$ layer taken from the glass substrate side of an AgNW/$MAPbI_3$ layer a) before and b) after the MA gas treatment.

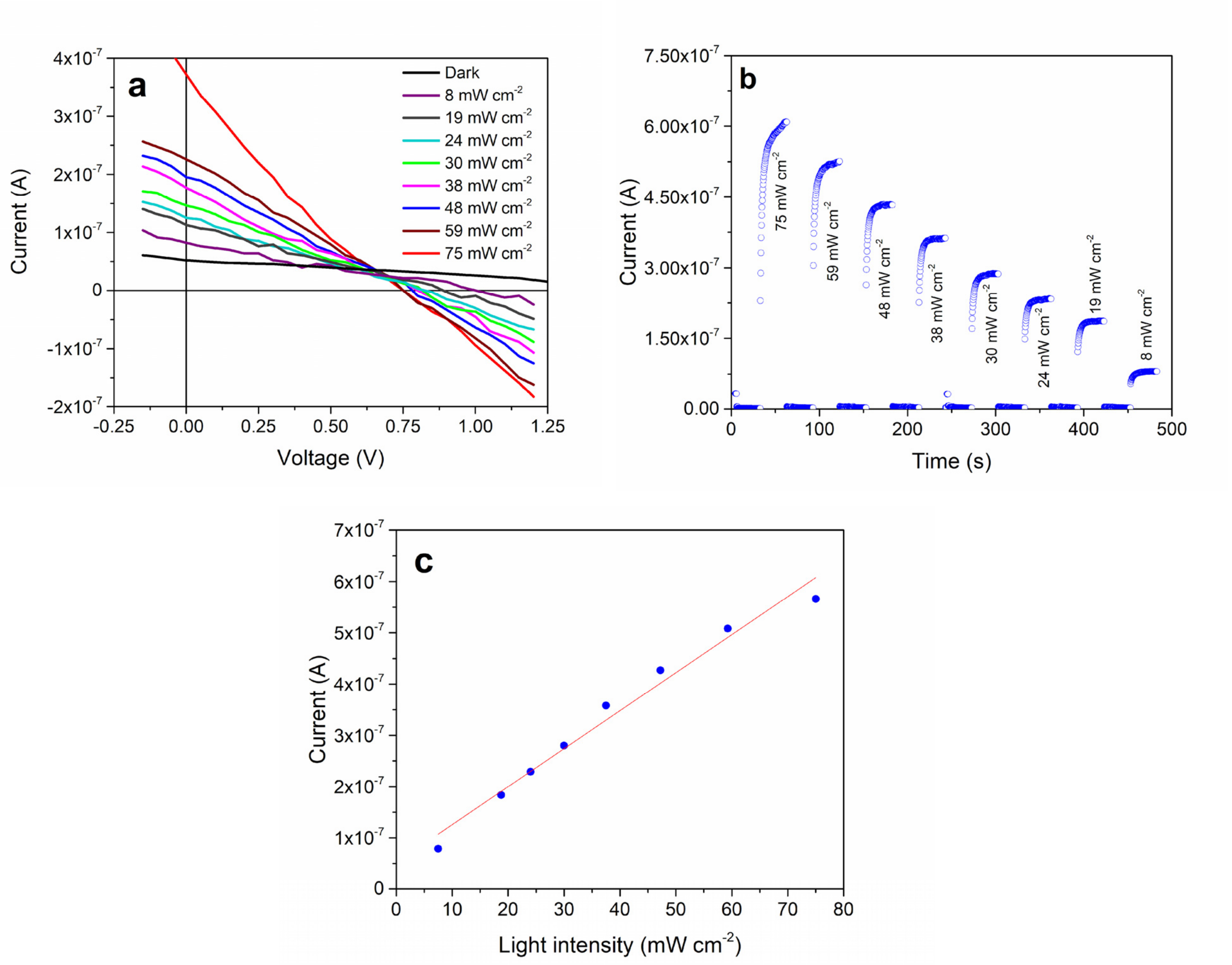

Figure S6. a) *I-V* curves of MSM PPDs with embedded AgNW network electrodes. b) *I-t* curve of MSM PPDs with embedded AgNW network electrodes, measured under zero bias and flashing white LED light with varying intensity. c) Photocurrent vs. intensity plot obtained from the analysis of the *I-t* curve shown in b).

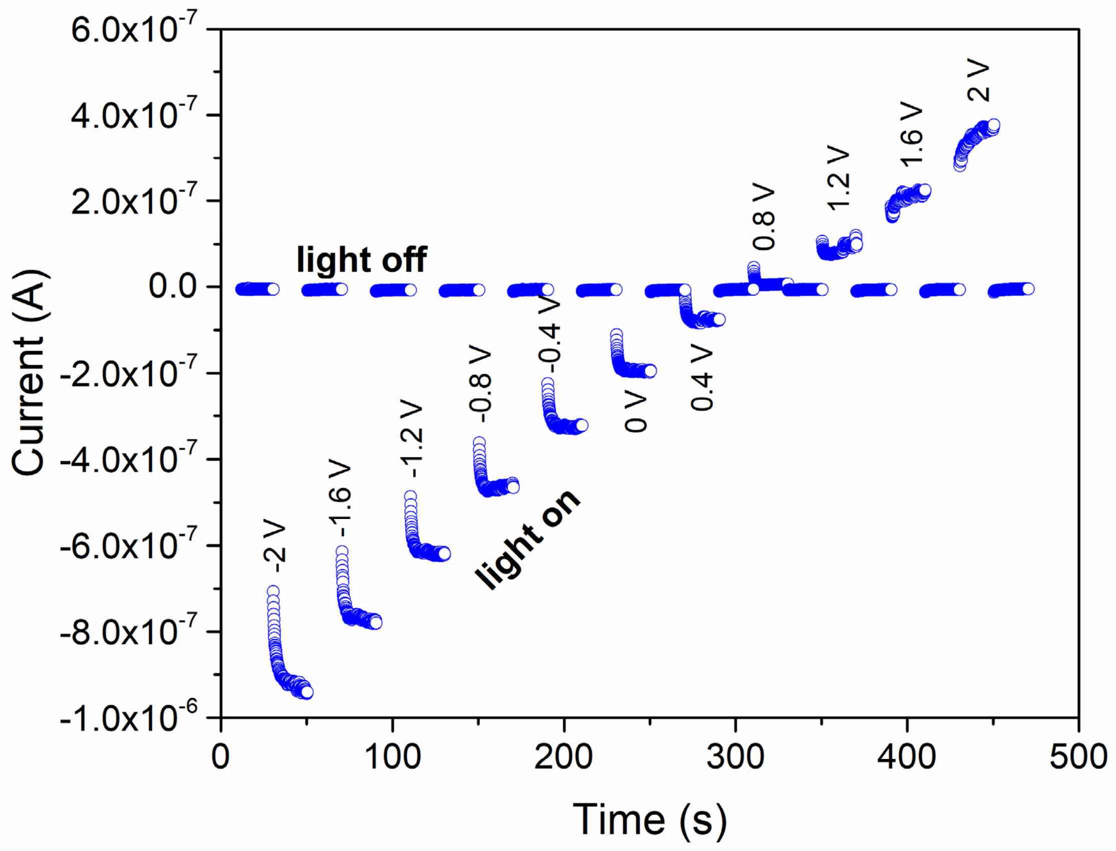

Figure S7. *I-t* curve of MSM PPDs with the embedded AgNW network electrode, measured under different applied bias and flashing white LED light with a constant intensity (75 mW cm^{-2}).

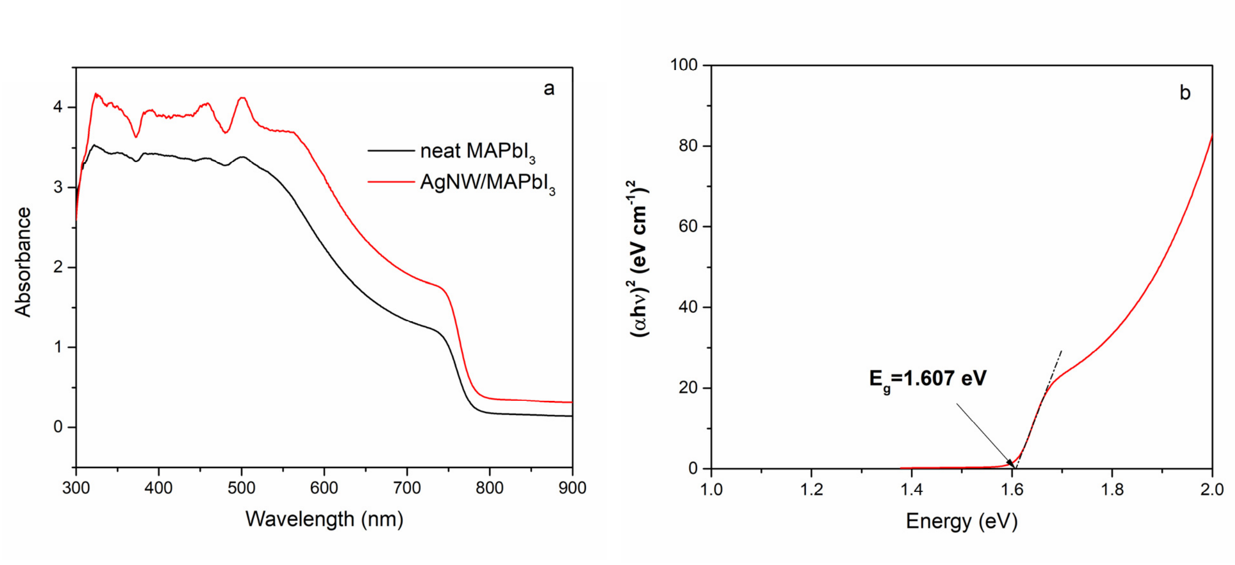

Figure S8. a) UV-Vis absorbance spectra of $MAPbI_3$ and AgNW/$MAPbI_3$ films; b) Tauc plot for neat $MAPbI_3$.

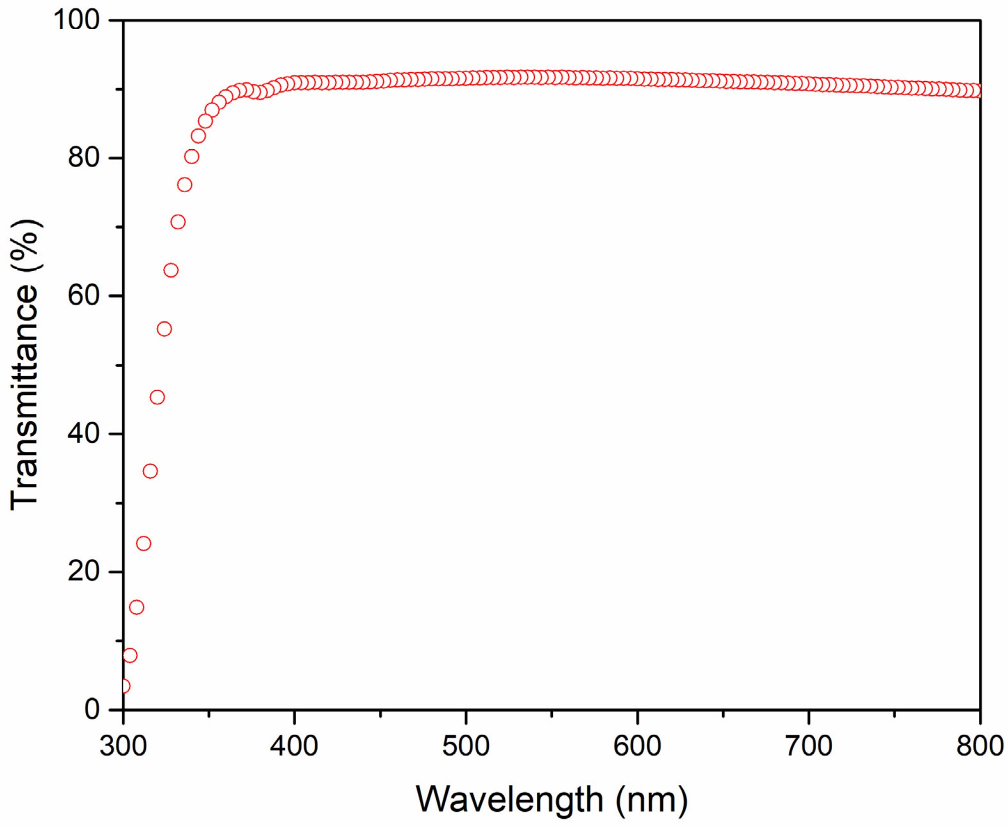

Figure S9. UV-Vis transmittance spectrum of soda-lime glass substrate.

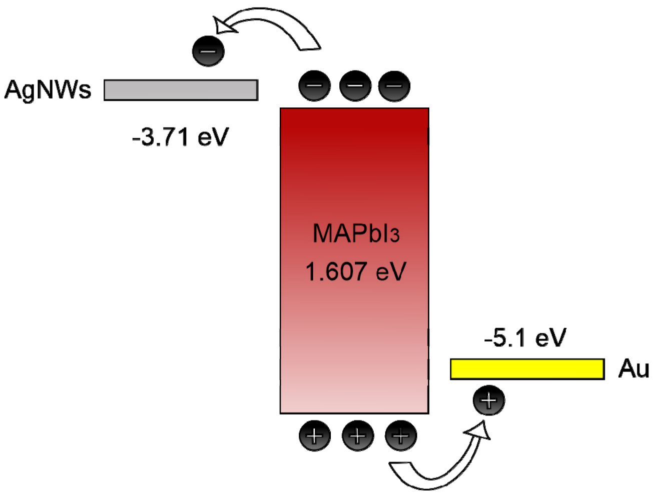

Figure S10. Band diagram for MSM PPD device.
